# HO-1 Protects against Hypoxia/Reoxygenation-Induced Mitochondrial Dysfunction in H9c2 Cardiomyocytes

**DOI:** 10.1371/journal.pone.0153587

**Published:** 2016-05-03

**Authors:** Dongling Chen, Zhe Jin, Jingjing Zhang, Linlin Jiang, Kai Chen, Xianghu He, Yinwei Song, Jianjuan Ke, Yanlin Wang

**Affiliations:** 1 Department of Anesthesiology, Zhongnan Hospital of Wuhan University, Wuhan, China; 2 Department of Ophthalmology, Tongji Hospital, Tongji Medical College, Huazhong University of Science and Technology, Wuhan, China; National Institutes of Health, UNITED STATES

## Abstract

**Background:**

Mitochondrial dysfunction would ultimately lead to myocardial cell apoptosis and death during ischemia-reperfusion injuries. Autophagy could ameliorate mitochondrial dysfunction by autophagosome forming, which is a catabolic process to preserve the mitochondrial’s structural and functional integrity. HO-1 induction and expression are important protective mechanisms. This study in order to investigate the role of HO-1 during mitochondrial damage and its mechanism.

**Methods and Results:**

The H9c2 cardiomyocyte cell line were incubated by hypoxic and then reoxygenated for the indicated time (2, 6, 12, 18, and 24 h). Cell viability was tested with CCK-8 kit. The expression of endogenous HO-1(RT-PCR and Western blot) increased with the duration of reoxygenation and reached maximum levels after 2 hours of H/R; thereafter, the expression gradually decreased to a stable level. Mitochondrial dysfunction (Flow cytometry quantified the ROS generation and JC-1 staining) and autophagy (The Confocal microscopy measured the autophagy. RFP-GFP-LC3 double-labeled adenovirus was used for testing.) were induced after 6 hours of H/R. Then, genetic engineering technology was employed to construct an Lv-HO1-H9c2 cell line. When HO-1 was overexpressed, the LC3II levels were significantly increased after reoxygenation, p62 protein expression was significantly decreased, the level of autophagy was unchanged, the mitochondrial membrane potential was significantly increased, and the mitochondrial ROS level was significantly decreased. Furthermore, when the HO-1 inhibitor ZnPP was applied the level of autophagy after reoxygenation was significantly inhibited, and no significant improvement in mitochondrial dysfunction was observed.

**Conclusions:**

During myocardial hypoxia-reoxygenation injury, HO-1 overexpression induces autophagy to protect the stability of the mitochondrial membrane and reduce the amount of mitochondrial oxidation products, thereby exerting a protective effect.

## Introduction

To date, mitochondria have been thought to play an important role in myocardial ischemia-reperfusion (I/R) injury [1]. It is well known that mitochondria, which provide the energy and biological oxidative substrates required for cell survival via oxidative phosphorylation, are indispensable in maintaining the function of myocardial cells [2]. Currently, studies have shown that energy depletion, oxidative stress, degraded protein fragments, and damaged organelles can also trigger autophagy [3], a catabolic process by which damaged cytoplasmic proteins and organelles are degraded via a lysosome-dependent mechanism that protects the cell.

In contrast, heme oxygenase-1 (HO-1), which belongs to the low-molecular weight heat shock protein HSP family, induction and expression are important protective mechanisms during cell stress [4–6]. In rats, it is believed that five main mechanisms are responsible for the myocardial protection of the HO-1 system: an anti-oxidative mechanism [7]; the maintenance of microcirculation [8]; and anti-apoptosis [9], anti-inflammation [10,11] and anti-arrhythmia [12] mechanisms. Moreover, some studies of HO-1 activation and inhibition have shown that HO-1 exerts an anti-apoptotic effect via its catalytic reaction product CO. Accordingly, HO-1 can prevent I/R injury during heart transplant surgery [13]. To date, many studies on the impact of HO-1 on post-ischemia-reperfusion cell autophagy and mitochondrial damage have focused on animal models of cardiac injury [14], lung injury [15] and liver injury [16–19]. In recent years, we used a rat model transduced with a PEP-HO-1 fusion protein (an exogenous cell-penetrating peptide [PEP] fused with HO-1) and studied the anti-inflammatory and anti-apoptotic effects of PEP-HO-1 on cardiac ischemia-reperfusion injury [20], intestinal ischemia-reperfusion injury [21] and distant organ damage resulting from intestinal ischemia-reperfusion injury [22]. However, the role of HO-1 in mitochondrial dysfunction which induced by I/R injury is still unknown.

Therefore, in this study, we chose to use genetic engineering techniques to construct an Lv-HO1-H9c2 cell line that enables a steady increase in the expression of exogenous HO-1. After myocardial hypoxia-reoxygenation injury, the RFP-GFP-LC3 double-labeled adenovirus was used to detect autophagy, and flow cytometry was used to measure mitochondrial membrane potential and mitochondrial ROS levels to investigate the mechanism by which HO-1 protects mitochondrial function following myocardial hypoxia-reoxygenation injury.

## Results

### 1. Effects of hypoxia/reoxygenation on cell viability and HO-1 expression levels in H9c2 cells

**Fig 1:**
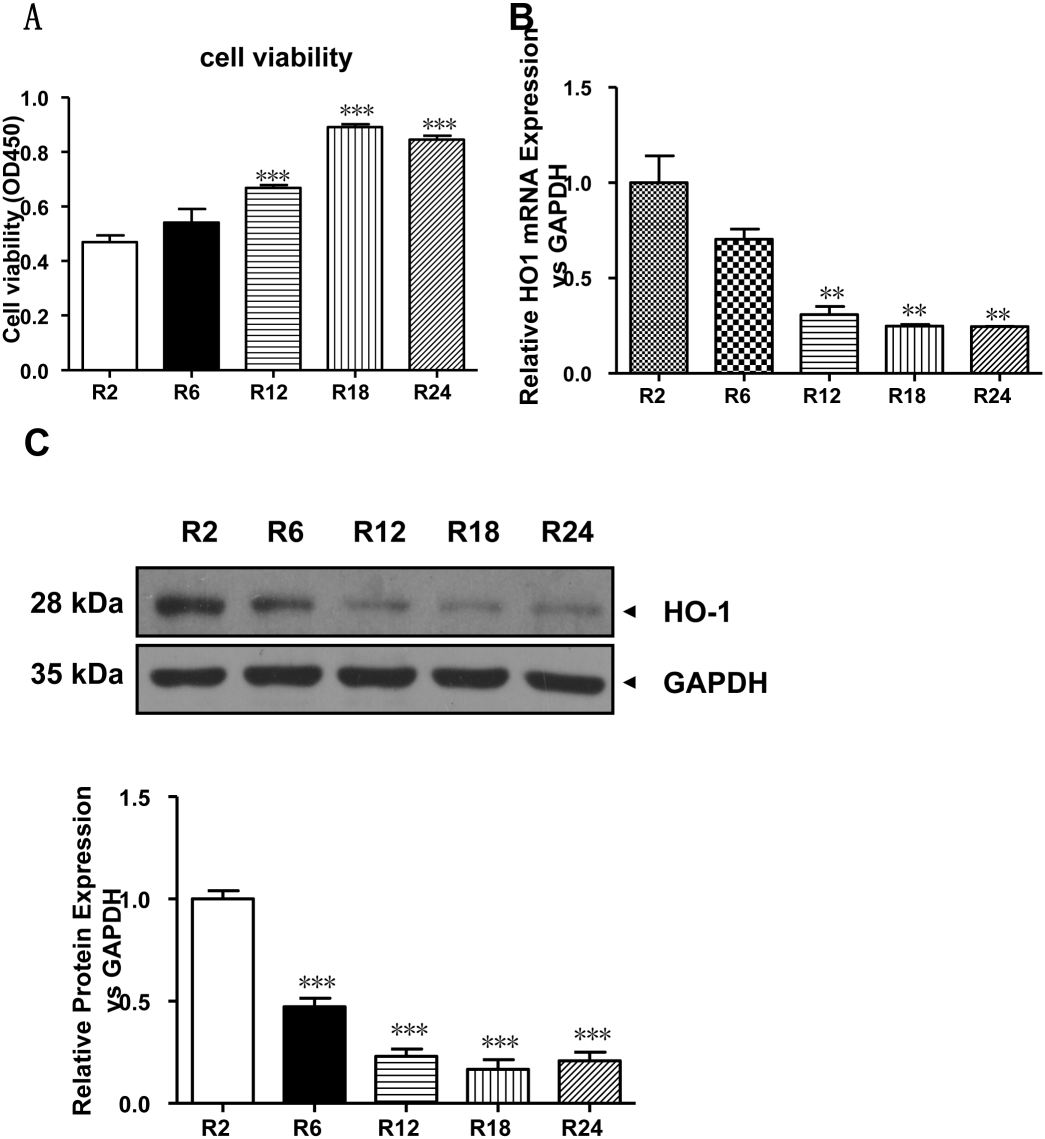
Effects of hypoxia/reoxygenation on cell viability and HO-1 expression levels in H9c2 cells. (A) H/R-induced cytotoxicity in H9c2 cells. Cell viability was measured using the CCK-8 assay. The data are presented as means ± SE (N = 5). ***p<0.001 vs. the normoxia group. (B) Real-time quantitative PCR (RT q-PCR) of HO-1 mRNA expression in H9c2 cells subjected to H/R for the indicated time relative to GAPDH expression (n = 3 wells per group). **p<0.01 vs. the normoxia group. (C) Time course of HO-1 protein expression analyzed using western blots of H9c2 cells. The expression of GAPDH was used as a loading control (n = 3 wells per group). *** p<0.001 vs. the normoxia group. H/R, Hypoxia/reoxygenation; HO-1, heme oxygenase-1.

The effect of different durations of hypoxia/reoxygenation on myocardial cytotoxicity was measured. As shown in Fig 1A, H9c2 cells were subjected to different durations of reoxygenation (2, 6, 12, 18, and 24 h). At 2 hours of reoxygenation, cell viability was at a minimum, and cytoxicity damage was greatest; however, by 18 hours of hypoxia-reoxygenation, cell viability had gradually stabilized. Significant differences occurred between the 12-, 18- and 24-hour reoxygenation groups and the 2-hour reoxygenation group (***p < 0.001, vs. the normoxia group). At 6 hours of reoxygenation, the extent of damage was approximately 40%; RT-PCR (Fig 1B) and Western Blotting (Fig 1C) were used to detect endogenous HO-1 expression in un-transduced H9c2 cells; HO-1 expression levels were highest at 2 hours of reoxygenation and were significantly lower at 12, 18 and 24 hours of reoxygenation (**p < 0.01, vs. the normoxia group). The HO-1 protein expression level decreased over time after reoxygenation and remained at a stable, low level at 12 hours of reoxygenation.

### 2. Hypoxia/reoxygenation-induced mitochondrial dysfunction and autophagy level in H9c2 cells

**Fig 2:**
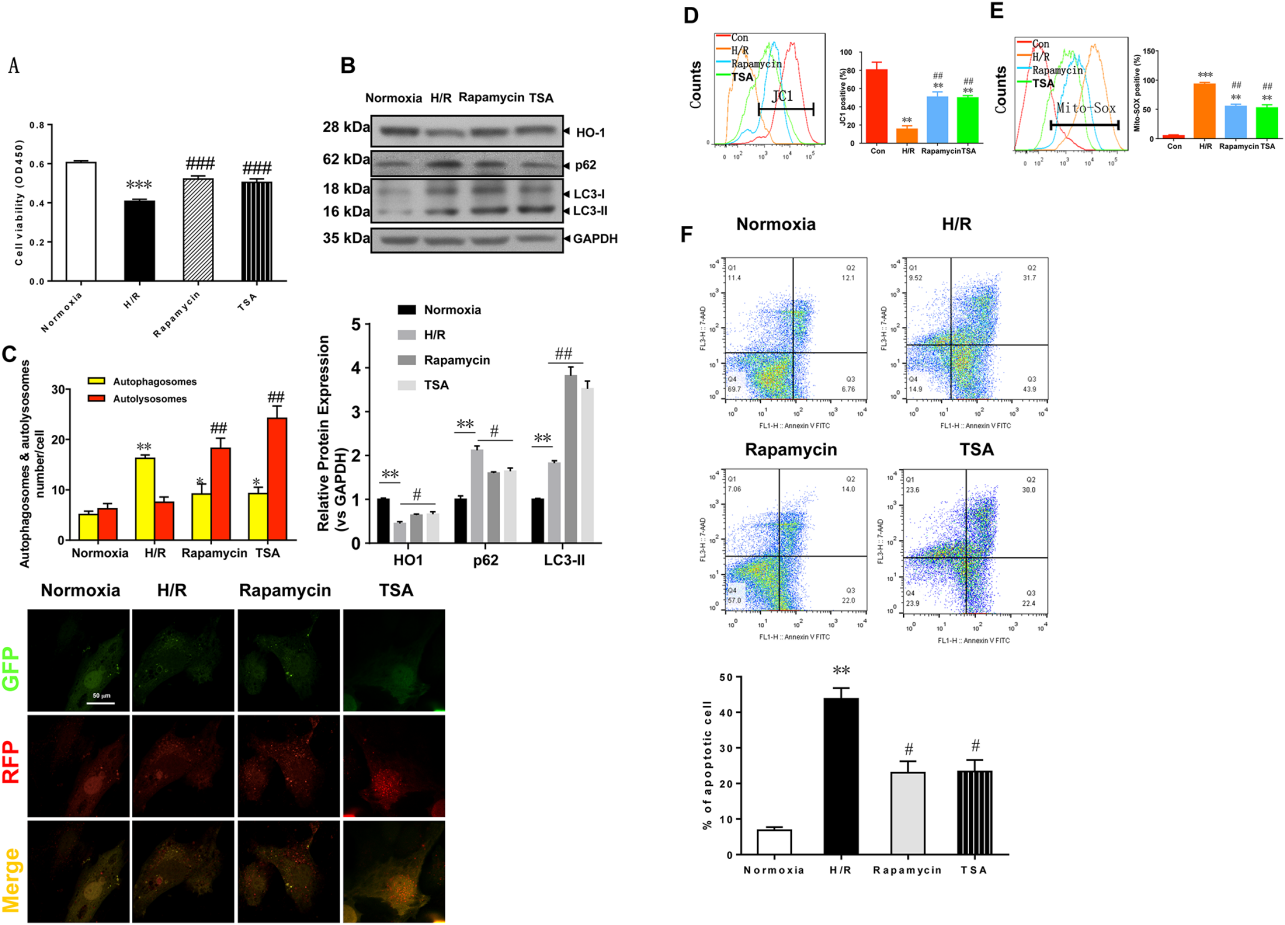
Hypoxia/reoxygenation-induced mitochondrial dysfunction and autophagy level in H9c2 Cells. (A) Cell viability was measured using the CCK-8 assay. The data are presented means ± SE (N = 5). ***p<0.001 vs. normoxia group. ###p<0.001 vs. corresponding H/R group. (B) H/R induced HO-1, p62, and LC-3 protein expression analyzed using western blots of H9c2 cells. GAPDH was used as a loading control (n = 3 wells per group). *p<0.05 vs. corresponding normoxia group; #p<0.05, vs. corresponding H/R group. (C) Representative confocal microscopy images and quantitative analysis of autophagosomes from 15 fields (n = 3 hearts per group). Scale bar = 500 nm. **p<0.01 vs. corresponding normoxia group; ##p<0.01 vs.corresponding H/R group. H/R, Hypoxia/reoxygenation; HO-1, heme oxygenase-1. (D,E) Flow cytometry detection of changes in JC-1 fluorescence color reflects changes in the mitochondrial membrane potential and mitochondrial ROS levels. **p<0.01 vs. corresponding normoxia group; ***p<0.001 vs corresponding Normoxia group. ##p<0.01, vs corresponding H/R group. (F) The apoptosis rate of the four groups. Cell identification and detection of apoptosis. **p<0.01 vs corresponding Normoxia group; #p<0.05, vs corresponding H/R group.

Cell viability was compared between the four groups (Fig 2A). Significant differences were found between the normoxic group and the H/R group (*** p < 0.001, vs corresponding Normoxia group) and between the H/R group and the rapamycin/TSA group (### p < 0.001, vs corresponding H/R group). Thus, pre-treatment with the autophagy inducer rapamycin or TSA inhibited H/R-induced damage, as demonstrated by the results obtained at 6 hours of reoxygenation. The expression levels of HO-1, p62 and LC3-II in the four groups were also compared (Fig 2B). HO-1 expression was higher and p62 and LC3-II expression was lower in the normoxic group. In the H/R group, HO-1 expression was significantly lower (*p<0.05, vs corresponding Normoxia group), p62 expression was significantly higher(*p<0.05, vs corresponding Normoxia group), and LC3-II expression was also higher(*p<0.05, vs corresponding Normoxia group). After rapamycin or TSA induction, the use of autophagic markers, LC3II expression was significantly increased (#p<0.05, vs corresponding H/R group), p62 expression was significantly decreased (#p<0.05, vs corresponding H/R group), and autophagy was relatively constant. Moreover, rapamycin or TSA increased levels of HO-1 (#p<0.05, vs corresponding H/R group). Fig 2C shows changes in autophagy in the four groups. In the H/R group, there was significant increase in yellow fluorescence (* p < 0.05 vs. the corresponding normoxia group), indicating impeded autophagy. In the rapamycin and TSA group, there was significant increase in red fluorescence, indicating unimpeded autophagy (## p < 0.01 vs. the corresponding H/R group). During hypoxia-reoxygenation, autophagy occurred with impeded autophagy, and the expression levels of p62 and LC3-II were significantly increased. Fig 2D and 2E shows the effect of hypoxia-reoxygenation on mitochondrial function. After myocardial hypoxia-reoxygenation, the mitochondrial membrane potential was significantly reduced (**p<0.01 vs corresponding Normoxia group), and mitochondrial ROS levels were significantly higher (***p<0.001 vs corresponding Normoxia group). After the use of autophagy markers, the level of autophagy was increased, the mitochondrial membrane potential was significantly increased (##p <0.01 vs. corresponding H/R group), and mitochondrial ROS levels were significantly decreased (**p<0.01, vs corresponding Normoxia group, ##p<0.01, vs corresponding H/R group). These results show that autophagy contributed to mitochondrial membrane stability and the generation of mitochondrial ROS. Fig 2F shows the apoptosis rate of the four groups. In H/R group, the apoptosis rate increased significantly (** p <0.01 vs. corresponding Normoxia group), while in the Rapamycin and TSA group, apoptosis rate compared with the H/R group, there was significantly reduced (#p <0.05, vs. corresponding H / R group).

### 3. Effects of HO-1 overexpression on mitochondrial dysfunction and autophagy level in Lv-HO-1-H9c2 cells with H/R model

**Fig 3:**
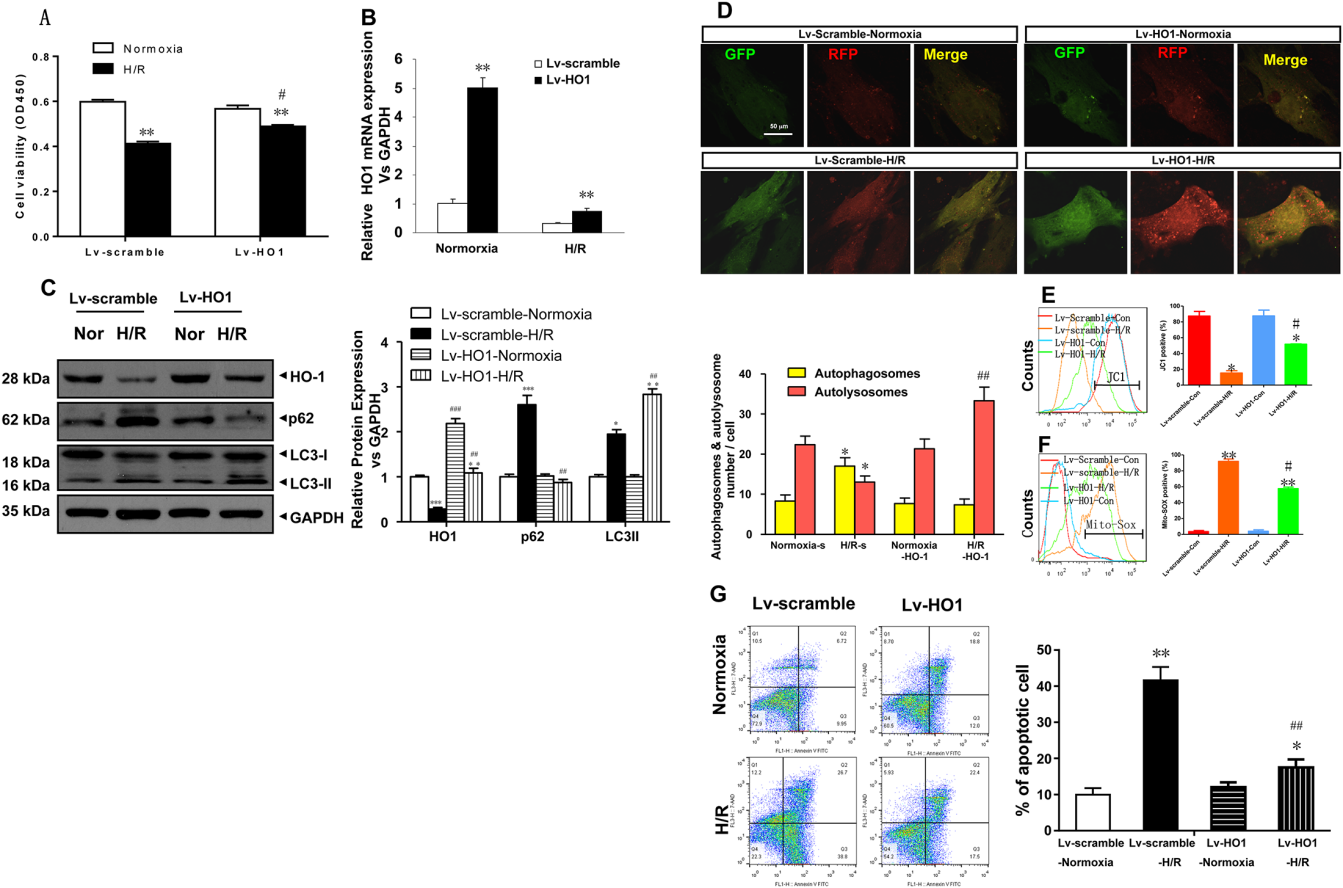
Effects of HO-1 overexpression on mitochondrial dysfunction and autophagy level in Lv-HO-1-H9c2 cells with H/R model. (A) Cell viability was measured using the CCK-8 assay. The data are presented as means ± SE (N = 5). **p<0.01 vs corresponding Normoxia group; #p<0.05, vs corresponding Lv-scramble group. (B) Real-time quantitative PCR (RT q-PCR) analyses of HO-1 mRNA expression in lv-HO-1 H9c2 cells subjected to H/R relative to GAPDH expression (n = 3 wells per group). **p<0.01 vs. the normoxia group. (C) H/R-induced HO-1, p62, and LC-3 protein expression analyzed using western blots of lv-HO-1 H9c2 cells. GAPDH was used as a loading control (n = 3 wells per group). ##p<0.01 vs corresponding Lv-scramble group; ***p<0.001 vs. the corresponding normoxia group. (D) Representative confocal microscopy images and quantitative analysis of autophagosomes from 15 fields (n = 3 hearts per group). Scale bar = 500 nm. *p<0.05 vs. the corresponding normoxia group; ##p<0.01, vs corresponding Lv-scramble group. H/R, Hypoxia/reoxygenation; HO-1, heme oxygenase-1. (E,F) Flow cytometry detection of changes in JC-1 fluorescence color reflects changes in the mitochondrial membrane potential and mitochondrial ROS levels. #p<0.05, vs corresponding Lv-scramble group; **p<0.01 vs corresponding Normoxia group, #p<0.05, vs corresponding Lv-scramble group. (G) The apoptosis rate of the four groups. Cell identification and detection of apoptosis. **p<0.01 vs corresponding Normoxia group; ##p<0.01, vs corresponding Lv-scramble group.

Cell viability was compared between the four groups (Fig 3A). An Lv-HO-1-H9c2 cell line encoding lentivirus-mediated HO-1 overexpression was constructed. HO-1 overexpression in H9C2 cells significantly inhibited H/R-induced cell injury (**p<0.01 vs corresponding Normoxia group, #p<0.05, vs corresponding Lv-scramble group). Significant HO-1 overexpression was observed in the Lv-HO1-H9c2 cell line (Fig 3B) (5-fold, **p<0.01 vs corresponding Normoxia group), suggesting that the Lv-HO1 group and the Lv-scramble group significantly differed under normoxic conditions. After reoxygenation, the expression level of HO-1 mRNA was significantly reduced but remained significantly higher than that in the normoxic group, suggesting that hypoxia/reoxygenation significantly reduced the stability of HO-1mRNA, even in the case of HO-1 overexpression. The expression levels of HO-1, p62, and LC3-II in each group were compared (Fig 3C). Under normoxic conditions, HO-1 expression was higher and LC-3 and p62 expression was lower in the Lv-HO1 group than in the Lv-scramble group (**p<0.01 vs corresponding Normoxia group). Under H/R conditions, with HO-1 over-expression, HO-1 expression was increased, LC-3 expression was increased, and p62 expression was decreased, suggesting that autophagy was unimpeded (##p<0.01, vs corresponding Lv-scramble group). Fig 3D shows changes in autophagy in the four groups. For the Lv-scramble cell line, yellow fluorescence was significantly increased and red fluorescence was significantly decreased in the H/R group compared with the normoxic group (*p<0.05 vs corresponding Normoxia group), indicating impeded autophagy. Under H/R conditions, red fluorescence was significantly increased in the Lv-HO1 group compared with the Lv-scramble group (##p<0.01, vs corresponding Lv-scramble group), indicating unimpeded autophagy. During hypoxia-reoxygenation, autophagy was impeded, and the expression of p62 and LC3-II was significantly increased. These results suggest that HO-1 overexpression contributed to the level of autophagy and inhibited H/R-induced cell injury. In the Lv-HO1-H9c2 cell line, the exogenous HO-1 expression level was increased (Fig 3E and 3F). After H/R treatment, the mitochondrial membrane potential was significantly increased (#p <0.05 vs. the corresponding H/R group) and the mitochondrial ROS level was significantly decreased (**p<0.01 vs corresponding Normoxia group, #p<0.05, vs corresponding Lv-scramble group), suggesting that HO-1 increased the mitochondrial membrane potential and maintained the stability of mitochondria while reducing mitochondrial ROS levels to reduce hypoxia-reoxygenation-induced oxidative stress. Fig 3G shows the apoptosis rate of the four groups. In Lv-scramble-H/R group, the apoptosis rate increased significantly (** p <0.01 vs. corresponding Normoxia group) when compared with Lv-scramble-Normoxia group, while in the Lv-HO1- H/R group, apoptosis rate compared with the Lv-scramble-H/R group, there was significantly reduced (##p<0.01, vs corresponding Lv-scramble group). It means that HO-1overexpression could reduce the rate of cell apoptosis to protect the cells damages.

### 4. Effects of the HO-1 inhibitor ZnPP on mitochondrial dysfunction and autophagy level in H9c2 cells with H/R model

**Fig 4:**
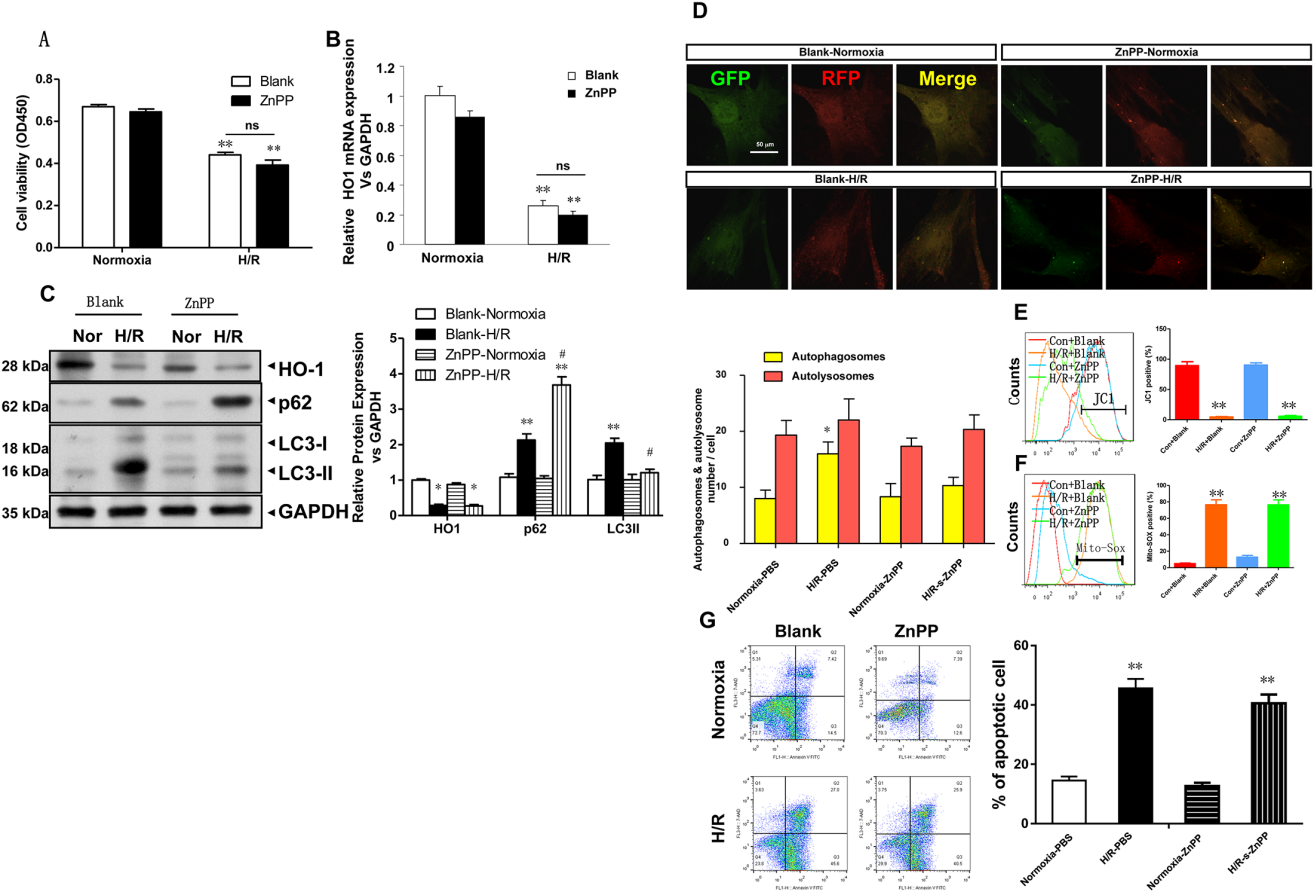
Effects of the HO-1 inhibitor ZnPP on mitochondrial dysfunction and autophagy level in H9c2 cells with H/R model. (A) Cell viability was measured using the CCK-8 assay. The data are presented as means ± SE (N = 5). **p<0.01, vs corresponding Normoxia group. (B) Real-time quantitative PCR (RT q-PCR) of HO-1 mRNA expression from H9c2 cells subjected to the HO-1 inhibitor ZnPP relative to GAPDH expression (n = 3 wells per group). **p<0.01, vs corresponding Normoxia group. (C) H/R induced HO-1, p62, and LC-3 protein expression analyzed using western blots of H9c2 cells subjected to the HO-1 inhibitor ZnPP. GAPDH was used as a loading control (n = 3 wells per group). *p<0.05 vs corresponding Normoxia group; #p<0.05, vs corresponding H/R group. (D) Representative confocal microscopy images and quantitative analysis of autophagosomes from 15 fields (n = 3 hearts per group). Scale bar = 500 nm. **p<0.01 vs corresponding Normoxia group. (E,F) Flow cytometry detection of changes in JC-1 fluorescence color reflects changes in the mitochondrial membrane potential and mitochondrial ROS levels. **p<0.01 vs. the corresponding normoxia group. (G) The apoptosis rate of the four groups. Cell identification and detection of apoptosis. **p<0.01 vs corresponding Normoxia group.

Cell viability in the four groups was compared (Fig 4A). After ZnPP was used to inhibit HO-1 activity, H/R induced cell injury became more severe; however, the difference was not statistically significant. Under H/R induced damages, the ZnPP inhibitor did not significantly alter the expression of HO-1 mRNA in the normoxia and H/R groups (Fig 4B). The expression levels of HO-1, p62 and LC3-II in the four groups were compared (Fig 4C). ZnPP was used to inhibit HO-1 activity; consequently, HO-1 expression was decreased, LC-3 expression was decreased, and p62 expression was increased, indicating impeded autophagy. Moreover, after ZnPP was used to inhibit HO-1 activity, there was no significant change in cell viability; however, the level of autophagy was significantly inhibited. After reoxygenation, LC3II expression was significantly lower than in the control group, whereas p62 expression was significantly higher, indicating impeded autophagy. These results show that the inhibition of HO-1 led to the significant inhibition of autophagy. Changes in autophagy in the four groups are shown in Fig 4D. In the H9c2 cell line, yellow fluorescence was significantly higher in the H/R group compared with the normoxic group (**p<0.01 vs corresponding Normoxia group), indicating impeded autophagy. Under H/R conditions, red fluorescence was slightly increased, but the difference was not statistically significant, indicating impeded autophagy. Fig 4D and 4E show that after HO-1 was inhibited by ZnPP, the mitochondrial membrane potential was significantly decreased (** p <0.01 vs. the corresponding normoxia group), and the mitochondrial ROS level was significantly increased (** p <0.01 vs. the corresponding normoxia group), suggesting that HO-1 inhibition did not improve H/R-induced mitochondrial dysfunction. Fig 4G shows the apoptosis rate of the four groups. In ZnPP-H/R group, the apoptosis rate increased significantly (** p <0.01 vs. corresponding Normoxia group).

## Discussion

HO is the initial and rate-limiting enzyme that catalyzes the oxidative metabolism of heme. Three HO isozymes exist, and HO-1 is the only isoenzyme that exhibits inductive expression. During cardiac ischemia-reperfusion injury, ROS generation can increase the expression of HO-1 [23], consistent with the results obtained in this study, and the expression of endogenous HO-1 increases with the duration of reoxygenation and reaches maximum levels after 2 hours of H/R; thereafter, the expression gradually decreases to a stable level over time (Fig 1B and 1C).

The autophagic dysfunction of myocardial cells has attracted attention as one of the mechanisms underlying ischemia-reperfusion injury [3,24,25]. In this study, autophagy was induced at 6 hours of H/R (Fig 2B and 2C); thereafter, the level of autophagy increased over time. After 12 hours of H/R, cell injury was gradually reversed because the level of myocardial autophagy was sufficiently high to eliminate harmful substances in the cells (Fig 1A), and the HO-1 expression level was gradually decreased (Fig 1B and 1C). In our controlled experiment using an autophagy inducer, rapamycin (cell autophagy marker) induction was observed, LC3II levels were significantly increased, p62 levels were significantly decreased, autophagy was unimpeded, and red fluorescence was significantly increased (Fig 2C). Mechanistically, in the ischemic myocardium, autophagy is mediated by Beclin-1 and AMPK-dependent pathways [26,27].

In addition, in this study, flow cytometry detection showed that during autophagy, the mitochondrial membrane potential was significantly higher (Fig 2D) and the mitochondrial ROS level was significantly reduced (Fig 2E). During ischemia, increased peroxide levels, mitochondrial electron transfer dysfunction, and ROS accumulation caused protein denaturation, mitochondrial dysfunction [28], opening of the transition pore of the mitochondrial membrane channel [1], damage to the mitochondrial membrane integrity, a lack of adenosine triphosphate (ATP), calcium overload inside the cell, and "insufficient" autophagy [29]; these forms of damage ultimately lead to myocardial cell apoptosis and death [24].

Therefore, clearing damaged mitochondria is extremely important. Autophagy, the major degradation pathway involved in mitochondrial quality control, has been reported as a cellular adaptive response to oxidative stresses [30]. Previous studies have emphasized that mitochondria present damaged mitochondrial membranes to the autophagosome [31] to preserve their structural and functional integrity. Once the autophagosome is formed, degraded LC3-II binds to ubiquitin, such as p62, to form a dimer. Aggregation of this dimer will form a dense cluster, in which p62 binds to ATG family proteins via the autophagy chain FYVE-related protein (ALFY), ultimately leading to selective autophagy [31] and cytoprotection.

Interestingly, in our studies, rapamycin could up-regulate the expression of the HO-1 protein (Fig 2B). It is also difficult to determine whether autophagy exerts its protective role by inhibiting the degradation and maintaining the stability of the HO-1 protein or by inducing high levels of expression of the endogenous HO-1 protein and promoting autophagosome formation. Therefore, an in-depth study was conducted.

To define the role of HO-1, most studies have used methods such as employing adeno-associated viruses to achieve instantaneous HO-1 over-expression [5,14] or knock-out models to down-regulate HO-1 expression, which have indirectly shown that HO-1 exerts a protective effect on liver ischemia-reperfusion injury [16,32]. However, these studies could not substantiate the effectiveness of gene therapy for treating ischemia-reperfusion injury. In our studies, a new gene therapy approach using sustained endogenous HO-1 expression in vivo was successfully used to consolidate and strengthen HO-1 protection. Hence, we used genetic engineering technology to successfully construct an Lv-HO1-H9c2 cell line (Fig 3A), which was used as a research model in hypoxia-reoxygenation experiments.

With HO-1 overexpression, LC3II levels were significantly increased after reoxygenation, and p62 protein expression was significantly decreased (Fig 3C). Moreover, detection of autophagic cells showed that red fluorescence was significantly increased (Fig 3D), the mitochondrial membrane potential was significantly increased (Fig 3E), and mitochondrial ROS levels were significantly decreased (Fig 3F), suggesting that HO-1 overexpression increased the level of autophagy and protected mitochondrial function, thereby inhibiting H/R induced cell injury. Furthermore, HO-1 was inhibited using ZnPP, and the level of autophagy after reoxygenation was significantly inhibited (Fig 4C and 4D) with no significant improvement in mitochondrial dysfunction (Fig 4E and 4F). It appears that the HO-1 protein stabilizes the mitochondrial membrane potential and reduces the mitochondrial ROS level via autophagy, thereby protecting myocardial cells from H/R induced injury [33]. Many evidences have revealed that a potential link on autophagic dysfunction and a change in susceptibility to cell death. However, when and how a change in autophagic pathway acitivity is able to affect cell viability is still a problem. As we known, an ATP reduction by 30–50% decreases autophagic volume by 70%, suggesting that a minimal energetic threshold must be maintained for the cell to respond adequately through autophagy [34]. Therefore, an increasing autophagic activity is compatible with cellular metabolism and function, then preserving viability. And an impaired autophagic machinery is not compatible with cellular metabolism and function leading low metabolite levels. If the insult or stress is mild, there is no great pack on cell viability. If the insult or stress is severe, it would result in apoptotoc or necrotic cell death [35]. In this study, H9c2 cells underwent a mild stress, ATP is still sufficient to the cellular metabolite, the cell viability was preserved.

Recent studies have shown that an HO-1 activator can activate transcription factor EB (TFEB), a regulator that induces lysosome and autophagosome formation, to protect against LPS-induced oxidative stress in heart tissue [36]. Meanwhile, caspase-3-mediated apoptosis and autophagy pathways may also be another important mechanism [25]. Lin et al. demonstrated that during CNS dysfunction, HO-1 acts on the mitochondrial membrane by activating miR-16, miR-17 and miR-140, thereby inhibiting autophagy [37]. Quinsay et al. suggested that BNIP3 regulates mitochondria and autophagy by regulating the mitochondrial membrane potential [38]. The relationship between HO-1 and autophagy has been investigated in both vital organs and cultural cells. However, its precise role remains controversial. The study of Bolisetty shows that inducible HO-1 overexpression in cells delays the onset of autophagy and significantly inhibits reactive oxygen species generation and apoptosis during cisplatin-mediated renal injury [39]. However, A Recent study have indicated that high HO-1 expression could promote autophagy by inhibiting mTOR, and cells are more sensitive to injury when HO-1 expression was inhibited [40]. And other animal model studies report increased HO-1 expression improves cardiac and vascular dysfunction and protects against liver ischemia-reperfusion injury by promoting autophagy and subsequently decreasing oxidative stress [41]. Therefore, the relationship between HO-1 and autophagy may vary depending on different organs and cells, and even at different stages. In this study, HO-1 induced autophagy in cardiac cells. The possible mechanism may also depending on TOR pathway.To date, it remains unclear whether HO-1 acts on the mitochondrial membrane to exert its effect on ischemia-reperfusion injury and which proteins on the mitochondrial membrane interact with HO-1 to promote cell autophagy and protect mitochondrial function. Although we did not focus on this topic here, we plan to study this topic in the future.

In summary, we based our conclusions on evidence that showed that during myocardial hypoxia-reoxygenation injury, HO-1 overexpression induces autophagy to protect the stability of the mitochondrial membrane and reduce the amount of mitochondrial oxidation products, thereby exerting a protective effect against myocardial hypoxia-reoxygenation injury. By studying the molecular and regulatory mechanisms of mitochondrial function protection, we will obtain further knowledge regarding the mechanism underlying the development of ischemic heart disease, information that will aid in for the development of new disease prevention and treatment strategies.

## Materials and Methods

### 1. Materials

Dulbecco’s Modified Eagle’s Medium (DMEM) and other cell culture supplies, such as trypsinogen, were purchased from Invitrogen. Fetal calf serum was purchased from Gibco BRL (Grand Island, NY, USA). A Cell Counting Kit-8 was purchased from Dojindo (Dojindo Laboratories, China). HO-1 and pAbR rabbit antibody were purchased from Enzo Life Sciences Inc. (Ann Arbor, Mich., USA). p62 and LC-3 antibody were purchased from Abcam (USA), and GAPDH antibody was obtained from CST (USA). AntiRabbit was obtained from Sigma (USA). BSA was obtained from Invitrogen (USA). TritonX-100 was obtained from Sigma (USA). A JC-1 (5,5’,6,6’-tetrachloro-1,1’,3,3’-tetraethylbenzimidazolcarbocyanine iodide) Mitochondrial Membrane Potential Detection Kit was purchased from Beyotime (China). Confocal microscopy and imaging systems (ZEISS-Axio) were used at the Wuhan University School of Basic Medical Sciences.

Zinc protoporphyrin (ZnPP), a HO-1 inhibitor, was supplied by Sigma. The purity of ZnPP (≥95%) was determined by HPLC; the inhibitor was soluble in water and has the molecular formula C_34_H_32_N_4_O_4_Zn and a molecular weight of 626.03 g/mol.

### 2. Cell culture

The H9c2 cardiomyocyte cell line (rat embryonic cardiomyoblasts) was maintained in our laboratory and obtained from the American Type Culture Collection (ATCC, Manassas, VA, USA). The H9c2 cells were maintained in high-glucose DMEM supplemented with 10% v/v fetal calf serum at 37°C in a humidified atmosphere containing 5% CO_2_. The medium was replaced every 2–3 days, and the cells were subcultured or subjected to experimental procedures at 80–90% confluence. In all experiments, the cells were rendered quiescent by serum starvation for 24 h before treatment. For the H/R experiments, hypoxic conditions were created by incubating the cells in an anaerobic chamber equilibrated with 2.5% O_2_, 5% CO_2_ and 92.5% N_2_ at 37°C for 24 h. The cells were then reoxygenated under normoxic conditions in a humidified atmosphere (95% air/5% CO2) at 37°C for the indicated time (2, 6, 12, 18, and 24 h). Normoxic control cells were incubated at 37°C under 95% air/5% CO_2_. In the Rapamycin experiments, the cells were pretreated for 1 hour with the autophagy inducer Rapamycin (100 nM) and then subjected to hypoxia and reoxygenation. In the ZnPP experiments, the cells were pretreated for 6 hours with the HO-1 inhibitor ZnPP (10 μM) and then subjected to hypoxia and reoxygenation.

### 3. Lv-HO1 H9c2 cell line construction

**Fig 5:**
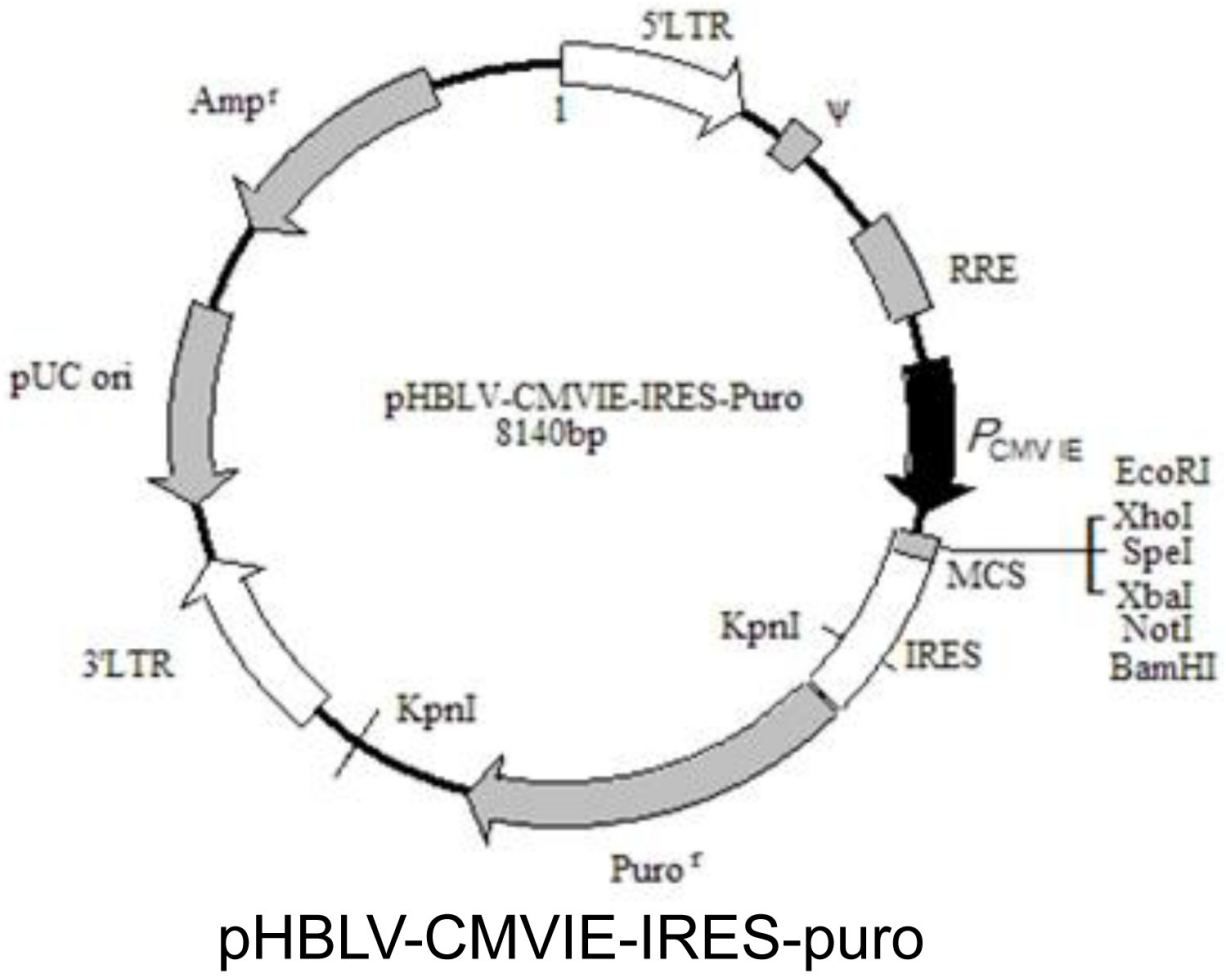
The vector map of pHBLV-CMVIE-IRES-puro. EcoRI and BamHI are insertion sites.

A lentiviral vector was constructed, and a lentiviral shuttle plasmid and its secondary packaging of the original vector plasmid were prepared; the three plasmid vectors were subjected to endotoxin-free extraction to obtain materials of high purity. Next, the plasmid vectors were co-transfected into 293T cells. The medium was replaced with complete medium at 6 hours after transfection. The supernatant, which was rich in lentivirus particles, was collected after 48 and 72 hours of culture and filtered using a 0.45 μm filter (Millipore), and the virus was then concentrated by ultracentrifugation. Next, a high-titer lentivirus concentrate was obtained after concentration. A vector map of pHBLV-CMVIE-IRES-puro is shown in Fig 5; EcoRI and BamHI are insertion sites.

The pHBLV-CMVIE-IRES-puro vector contains a CMVIE promoter to initiate the expression of target genes and contains a puromycin resistance gene as a marker. The HO-1primer designs as Table 1. The HO-1 target sequence is as follows:

ATGGAGCGTCCGCAACCCGACAGCATGCCCCAGGATTTGTCAGAGGCCCTGAAGGAGGCCACCAAGGAGGTGCACACCCAGGCAGAGAATGCTGAGTTCATGAGGAACTTTCAGAAGGGCCAGGTGACCCGAGACGGCTTCAAGCTGGTGATGGCCTCCCTGTACCACATCTATGTGGCCCTGGAGGAGGAGATTGAGCGCAACAAGGAGAGCCCAGTCTTCGCCCCTGTCTACTTCCCAGAAGAGCTGCACCGCAAGGCTGCCCTGGAGCAGGACCTGGCCTTCTGGTACGGGCCCCGCTGGCAGGAGGTCATCCCCTACACACCAGCCATGCAGCGCTATGTGAAGCGGCTCCACGAGGTGGGGCGCACAGAGCCCGAGCTGCTGGTGGCCCACGCCTACACCCGCTACCTGGGTGACCTGTCTGGGGGCCAGGTGCTCAAAAAGATTGCCCAGAAAGCCCTGGACCTGCCCAGCTCTGGCGAGGGCCTGGCCTTCTTCACCTTCCCCAACATTGCCAGTGCCACCAAGTTCAAGCAGCTCTACCGCTCCCGCATGAACTCCCTGGAGATGACTCCCGCAGTCAGGCAGAGGGTGATAGAAGAGGCCAAGACTGCGTTCCTGCTCAACATCCAGCTCTTTGAGGAGTTGCAGGAGCTGCTGACCCATGACACCAAGGACCAGAGCCCCTCACGGGCACCAGGGCTTCGCCAGCGGGCCAGCAACAAAGTGCAAGATTCTGCCCCCGTGGAGACTCCCAGAGGGAAGCCCCCACTCAACACCCGCTCCCAGGCTCCGCTTCTCCGATGGGTCCTTACACTCAGCTTTCTGGTGGCGACAGTTGCTGTAGGGCTTTATGCCATGTGA

**Table 1:** Primer design.

Name	Sequence
HO-1U	*EcoRI*
	CAGGAATTCATGGAGCGTCCGCAA
HO-1D	*BamHI*
	CAGGGATCCTCACATGGCATAAAGC

### 4. Cell viability

After H9c2 and Lv-HO1-H9c2 cells were cultured in 96-well plates and received the described treatments, 10 μl of CCK-8 solution was added to each well (1/10 dilution); the plates were then incubated for a further 3 h. The absorbance was measured at 450 nm using a microplate reader (SpectraMax M5 Microplate Reader, Molecular Devices, USA). The mean optical density (OD) of five wells in the indicated groups was used to calculate the percent cell viability according to the following formula: Percent cell viability = OD treatment group/OD control group×100%. The experiments were repeated 3 times.

### 5. Real-time quantitative PCR

Total RNA was isolated from cells using TRIzol Reagent (Invitrogen) and purified using the RNeasy Total RNA Isolation Kit (Invitrogen). Real-time quantitative PCR was performed using the ABI 7900 Real-Time PCR System (Applied Biosystems) and the SYBR^®^Premix Ex Taq^™^ Perfect Real-Time Kit. To measure mouse gene expression, the following SYBR Green real-time PCR primers were used:

HO-1: Forward 5′- TGCACATCCGTGCAGAGAAT -3′

    Reverse 5′-CTGGGTTCTGCTTGTTTCGC -3′; product length = 147

GAPDH: Forward 5′—CACCATCTTCCAGGAGCGAG -3′

    Reverse 5′—AAATGAGCCCCAGCCTTCTC -3′; product length = 114

### 6. Western blot analysis

Proteins were isolated from the cells that had been subjected to hypoxia and reoxygenation using standard protocols, and the protein concentrations in the lysates were determined using the Bradford Protein Assay Kit (Beyotime, China). Equal quantities of proteins (30–50 lg/lane) were subjected to 8%–12% SDS-PAGE, depending on the target proteins, electrotransferred onto nitrocellulose membranes, and incubated with primary antibodies against HO-1 (Enzo; 1:1,000), p62 (Abcam, 1:2,000), LC-3-II (Abcam, 1:1,500), GAPDH (CST, 1:4,000), and antiRabbit (Sigma, 1:8,000). After incubating the membranes with the corresponding secondary antibodies, protein bands were detected using a chemiluminescence imaging analysis system (Fujifilm), and quantitation was performed using Quantity One 4.4.0 software (Bio-Rad).

### 7. Confocal microscopy autophagy measurement

To 6-well plates (pre-covered with a small, thin glass sheet for cell adhesion), 2 mL of Lv-HO1-H9c2 cell suspension (density: 2 × 10^4^ cells/mL) was added. Cells were transfected with Ad-mCherry-LC3-GFP adenovirus (MOI = 50); 36 hours later, the plates were pre-incubated for 24 hours in an incubator (37°C, 5% CO_2_). Next, 4% PFA (paraformaldehyde) was used to fix the cells for more than 10 minutes; the cells were then washed in PBS (5 minutes x 3 times). Then, Antifade mounting medium was added, and the cells were imaged under a fluorescent microscope. RFP-GFP-LC3 double-labeled adenovirus was used for testing. GFP degrades in the acidic environment used. When red and green fluorescent images are merged, the yellow spots that appear indicate autophagosomes. Red spots indicate autophagic lysosomes. In normal phagosome-lysosome fusions, there will be more red fluorescence than yellow fluorescence. In impeded autophagy, where phagosome-lysosome fusion does not work, yellow fluorescence will be dominant.

### 8. Flow cytometry quantification of ROS generation

Mitochondrial Superoxide Indicator, as a novel mitochondrial fluorescent probes, can specifically target the mitochondria, thereby selectively detecting superoxide within mitochondria. After incubation with the MitoSOX staining solution at 37°C in the cell incubator for 10 min, cells were washed with MitoSOX staining buffer two times and then analyzed using a fluorescence microplate reader. The probe can live through the cell membrane and selectively enter the mitochondria. Once into mitochondria, the probe can be stained showing red fluorescence after superoxide oxidation. It can be super-oxide rather than other reactive oxygen species (ROS) and reactive nitrogen species (RNS) rapid oxidation. After binding nucleic acid oxidation products, the probe can produce large amounts of fluorescence by flow cytometry, thus, the changes of mitochondrial reactive oxygen species content may be reflected.

### 9. Detection of change in mitochondria membrane potential

Using flow cytometry to detect the 5,5',6,6'-tetrachloro-1,1',3,3'-. tetraethylbenzi-midazolylcarbocyanine iodide (JC-1) staining. After incubation with the JC-1 staining solution at 37°C in the cell incubator for 10 min, cells were washed with JC-1 staining buffer two times and then analyzed using a fluorescence microplate reader. In the normal mitochondria, JC-1 aggregate to form a polymer in the mitochondrial matrix, the polymer sends a strong red fluorescence (Ex = 585 nm, Em = 590 nm); While in the unhealthy mitochondrial, due to the decline or loss of the mitochondrial membrane potential, JC-1 monomers just can be present in the cytoplasm, resulting in a green fluorescence (Ex = 514 nm, Em = 529 nm). Therefore, using flow cytometry to observe the color changes reflects very directly the early change of mitochondrial membrane potential.

### 10. Detection of the rate of apoptosis

After treatment, 1~5×10^5^ cells were harvested and placed in EP tubes. The cells were rinsed with 0.1 mmol/L PBS (pH = 7.4) and collected brief centrifugation with trypsin EDTA-free. After that, the cells were resuspended in 500 μL of binding buffer, mixed with 5 μL of Annexin V-FITC, and then kept in darkness for 10 min at room temperature. Then, 5 μL of propidium iodide (PI) was added and the samples were kept in darkness for 10 min at room temperature. Approximately 300 μL of binding buffer was added, and the samples were analyzed using flow cytometry within 1 h. The blank control was the cells of the control group without Annexin V-FITC and PI. The Annexin V-FITC single-labeled control consisted of cells labeled with Annexin V-FITC. The PI single-labeled control consisted of cells labeled with PI.

### 11. Statistical analysis

The data are presented as means ± SEM. Statistical analysis was performed using the Mann-Whitney test for two-group comparisons. Significant differences between multiple treatments were calculated using one-way analysis of variance followed by the Bonferroni post hoc test when appropriate. Western blot densities were analyzed using the Kruskal-Wallis test followed by Dunn's post hoc test. Probabilities of 0.05 or less were considered statistically significant.

## Supporting Information

S1 Fig Effects of hypoxia/reoxygenation on cell viability and HO-1 expression levels in H9c2 cells. (A) Cell viability; (B) HO1 QPCR; (C)WB analysis.
(RAR)

S2 Fig Hypoxia/reoxygenation-induced mitochondrial dysfunction and autophagy level in H9c2 Cells. (A) Cell viability (CCK-8); (B) WB analysis; (C) Confocol-RFP-GFP-LC3; (D,E) Flow cytometry detection of changes in JC-1 and MitoSox; (F) apoptosis rate (AnnexinV-PI).
(RAR)

S3 Fig Effects of HO-1 overexpression on mitochondrial dysfunction and autophagy level in Lv-HO-1-H9c2 cells with H/R model. (A) Cell viability (CCK-8); (B) HO1 QPCR; (C) WB analysis; (D)Confocol-RFP-GFP-LC3; (E,F) Flow cytometry detection of changes in JC-1 and MitoSox; (G) apoptosis rate (AnnexinV-PI).
(RAR)

S4 Fig Effects of the HO-1 inhibitor ZnPP on mitochondrial dysfunction and autophagy level in H9c2 cells with H/R model. (A) Cell viability (CCK-8); (B) HO1 QPCR; (C) WB analysis; (D)Confocol-RFP-GFP-LC3; (E,F) Flow cytometry detection of changes in JC-1 and MitoSox; (G) apoptosis rate (AnnexinV-PI).
(RAR)
